# T2T reference genome assembly and genome-wide association study reveal the genetic basis of Chinese bayberry fruit quality

**DOI:** 10.1093/hr/uhae033

**Published:** 2024-01-30

**Authors:** Shuwen Zhang, Zheping Yu, Li Sun, Senmiao Liang, Fei Xu, Sujuan Li, Xiliang Zheng, Lijv Yan, Yinghong Huang, Xingjiang Qi, Haiying Ren

**Affiliations:** State Key Laboratory for Managing Biotic and Chemical Threats to Quality and Safety of Agro-products, Institute of Horticulture, Zhejiang Academy of Agricultural Sciences, 298 Desheng Road, Shangcheng District, Hangzhou 310021, Zhejiang, China; State Key Laboratory for Managing Biotic and Chemical Threats to Quality and Safety of Agro-products, Institute of Horticulture, Zhejiang Academy of Agricultural Sciences, 298 Desheng Road, Shangcheng District, Hangzhou 310021, Zhejiang, China; State Key Laboratory for Managing Biotic and Chemical Threats to Quality and Safety of Agro-products, Institute of Horticulture, Zhejiang Academy of Agricultural Sciences, 298 Desheng Road, Shangcheng District, Hangzhou 310021, Zhejiang, China; State Key Laboratory for Managing Biotic and Chemical Threats to Quality and Safety of Agro-products, Institute of Horticulture, Zhejiang Academy of Agricultural Sciences, 298 Desheng Road, Shangcheng District, Hangzhou 310021, Zhejiang, China; State Key Laboratory for Managing Biotic and Chemical Threats to Quality and Safety of Agro-products, Institute of Horticulture, Zhejiang Academy of Agricultural Sciences, 298 Desheng Road, Shangcheng District, Hangzhou 310021, Zhejiang, China; State Key Laboratory for Managing Biotic and Chemical Threats to Quality and Safety of Agro-products, Institute of Horticulture, Zhejiang Academy of Agricultural Sciences, 298 Desheng Road, Shangcheng District, Hangzhou 310021, Zhejiang, China; State Key Laboratory for Managing Biotic and Chemical Threats to Quality and Safety of Agro-products, Institute of Horticulture, Zhejiang Academy of Agricultural Sciences, 298 Desheng Road, Shangcheng District, Hangzhou 310021, Zhejiang, China; Linhai Specialty and Technology Extension Station, 219 Dongfang Avenue, Linhai 317000, Zhejiang, China; Jiangsu Taihu Evergreen Fruit Tree Technology Promotion Center, Dongshan Town, Wuzhong District, Suzhou 215107, Jiangsu, China; State Key Laboratory for Managing Biotic and Chemical Threats to Quality and Safety of Agro-products, Institute of Horticulture, Zhejiang Academy of Agricultural Sciences, 298 Desheng Road, Shangcheng District, Hangzhou 310021, Zhejiang, China; Xianghu Laboratory, 168 Gengwen Road, Xiaoshan District, Hangzhou 311231, Zhejiang, China; State Key Laboratory for Managing Biotic and Chemical Threats to Quality and Safety of Agro-products, Institute of Horticulture, Zhejiang Academy of Agricultural Sciences, 298 Desheng Road, Shangcheng District, Hangzhou 310021, Zhejiang, China

## Abstract

Chinese bayberry (*Myrica rubra* or *Morella rubra*; 2n = 16) produces fruit with a distinctive flavor, high nutritional, and economic value. However, previous versions of the bayberry genome lack sequence continuity. Moreover, to date, no large-scale germplasm resource association analysis has examined the allelic and genetic variations determining fruit quality traits. Therefore, in this study, we assembled a telomere-to-telomere (T2T) gap-free reference genome for the cultivar ‘Zaojia’ using PacBio HiFi long reads. The resulting 292.60 Mb T2T genome, revealed 8 centromeric regions, 15 telomeres, and 28 345 genes. This represents a substantial improvement in the genome continuity and integrity of Chinese bayberry. Subsequently, we re-sequenced 173 accessions, identifying 6 649 674 single nucleotide polymorphisms (SNPs). Further, the phenotypic analyses of 29 fruit quality-related traits enabled a genome-wide association study (GWAS), which identified 1937 SNPs and 1039 genes significantly associated with 28 traits. An SNP cluster pertinent to fruit color was identified on Chr6: 3407532 to 5 153 151 bp region, harboring two MYB genes (*MrChr6G07650* and *MrChr6G07660*), exhibiting differential expression in extreme phenotype transcriptomes, linked to anthocyanin synthesis. An adjacent, closely linked gene, *MrChr6G07670* (MLP-like protein), harbored an exonic missense variant and was shown to increase anthocyanin production in tobacco leaves tenfold. This SNP cluster, potentially a quantitative trait locus (QTL), collectively regulates bayberry fruit color. In conclusion, our study presented a complete reference genome, uncovered a suite of allelic variations related to fruit-quality traits, and identified functional genes that could be harnessed to enhance fruit quality and breeding efficiency of bayberries.

## Introduction

The genus *Myrica* L. encompasses approximately 55 species [1], including *Myrica rubra*, *Manihot esculenta*, *Myrica nana*, *Myrica adenophora*, *Myrica cerifera*, *Myrica faya*, and *Myrica rivas-martinezii*, distributed across Southeast Asia, North America, and Australia [2]. Of these, *M. rubra*, also known as Chinese bayberry or red bayberry, is a subtropical fruit species indigenous to southern China and the only *Myrica* species cultivated for economic purposes. Archeological evidence from the Neolithic Hemudu site suggests that cultivation dates back at least 7000 years [3]. *Myrica rubra* seedlings undergo an initial maturation period of up to ten years, eventually giving rise to dioecious evergreen trees with catkin inflorescence and a ZW sex-determination system [4, 5], thus making molecular-assisted breeding techniques crucial for seedling identification. Chinese bayberries are highly prized in China for their appealing color, distinctive sweet/sour flavor, nutritional content, and health benefits, including antibacterial, antioxidant, anti-inflammatory, and antitumor effects [6, 7].

Technological advancements in genomic sequencing and assembly have led to the acquisition of two sets of whole-genome sequence data for *M. rubra*. The initial genome assembly of the bayberry cultivar ‘Zaojia’ resulted in a 289.9 Mb scaffold genome (Zaojia Version 1.0) encoding 26 325 genes [8]. Subsequently, Jia et al. (2019) published Y2012–145 genomic data that provided a valuable resource for understanding plant sex chromosome evolution [4]. The genome sequence of bayberry has facilitated the discovery of allelic variation and functional genes associated with economically significant traits such as fruit quality. Nevertheless, these earlier published genomes were essentially framework genomes that lacked the comprehensive quality expected of true reference genomes, especially as there are a large number of gaps that affect sequence assembly continuity. These incomplete versions missed crucial regions, particularly centromeric and telomeric regions, and thus failed to achieve reference genome status. Such omissions directly impact the identification of functional genes and the integrity of the genome, affecting genetic research [9, 10]. Consequently, further refinement of these genomes is evidently needed. The compilation and dissemination of complete and accurate genomic sequences will enhance functional genomics and breeding research for this species.

**Figure 1:**
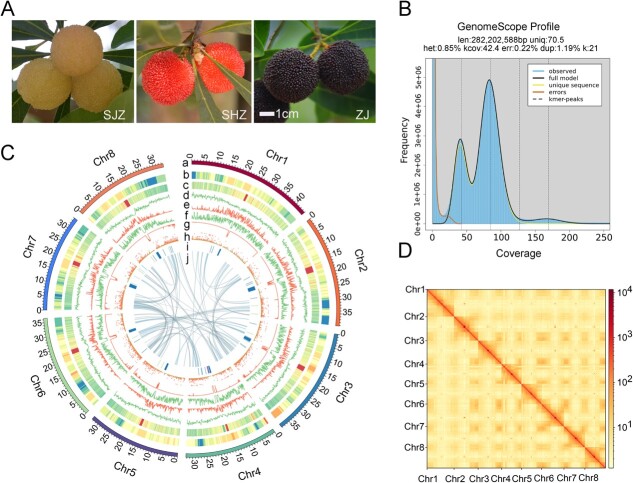
T2T Gap-Free Assembly of the Chinese Bayberry Reference Genome. A. Variations in the morphology of the bayberry fruit displayed by different colors: white (Shuijingzhong, SJZ), red (Shenhongzhong, SHZ), and black-purple (Zaojia, ZJ) fruit. Scale bars = 1 cm. B. K-mer distribution (17-mer) spectrum, illustrating the genome complexity of bayberry. C. Features and synteny of the bayberry genome arranged from the outermost (a) to innermost (j) rings: (a) chromosome length in megabases (Mb); (b) LAI per chromosome; (c) GC content, with darker shades indicating higher values; (d) gene density; (e) Gypsy element density; (f) Copia element density; (g) overall TE density; (h) gene expression levels in ZJ fruit, presented as fragments per kilobase of transcript per million mapped reads (FPKM) in 5-Mb windows, normalized with log_2_ (FPKM +1); (i) tandem repeat density associated with centromeres, highlighted in blue for higher densities; (j) syntenic relationships depicted with connecting lines between chromosomal regions. D. Hi-C interaction heatmap displaying the connections between the eight chromosomes, with darker colors representing higher interaction frequencies. The axes denote the sequential positions of the chromosomes.

Bayberries include several species with a wide distribution in southern China and a wealth of germplasm resources. For example, the fruit color varies from white to black-purple (Fig. 1A), and the species exhibits high genetic diversity within its germplasm [11, 12], which facilitates the elucidation of specific allelic variants associated with superior fruit quality. GWAS has high efficiency in site detection and precise gene localization. To date, however, large-scale resequencing of the available *M. rubra* germplasm resources has not been undertaken. Similar GWAS on other economically important crops such as tomato, grape, and loquat has been instrumental in identifying specific genes and mutations linked to fruit color, size, flesh texture, flavor, and nutrient/bioactive compound content [13, 14], aiding in understanding the genetic control of agronomic fruit traits and in the development of new molecular breeding methods. These studies have also provided methodological references for mining allelic variations that influence bayberry fruit quality.

Fruit quality is defined by a variety of parameters, including external quality traits such as shape, size, and color, as well as internal quality traits such as sugar, acidity, and amino acid levels [15, 16]. Therefore, fruit quality is a complex, multigenic trait that affects the commercial value of the fruit. Recent studies have identified several fruit quality-associated genes in bayberry, including *MrMYB1*, *MrMYB6*, *MrbHLH1*, *MrDFR1*, and *MrUFG1*[17–21]. *MrTPS3* and *MrTPS20* have been linked with β-caryophyllene and α-pinene production, respectively, contributing to the sweet taste of bayberries [22]. To date, analysis of quality-related loci in bayberry samples has relied on relatively inefficient methods such as homologous cloning, RACE amplification, or transcriptome sequencing, which has limited the breadth of target genes available for innovative molecular breeding [4, 5, 23], thus impeding the progress of bayberry breeding.

In this study, we reported the complete T2T, gap-free bayberry genome, Zaojia Version 2.0. We performed whole-genome re-sequencing on 167 cultivated germplasm resources and six interspecific resources. We then utilized integrated GWAS and transcriptomic sequencing analyses of extreme fruit phenotypes to identify allelic variants and candidate genes related to fruit quality. Our findings have the potential to advance the functional genomics development of Chinese bayberry plants through molecular breeding by effectively utilizing the available germplasm resources.

## Results

### Assembly of a highly contiguous genome of *M. Rubra*

Initially, approximately 19.21 GB of Pacific Biosciences (PacBio) HiFi reads (~65.66×) were generated (Tables S1 andS2). K-mer analysis estimated the *M. rubra* genome size to be 282.20 Mb with a heterozygosity rate of 0.85% (Table S3, Fig. 1B). A genome assembly of 292.60 Mb was ultimately produced, featuring a contig N50 size of 36.50 Mb and a GC content of 37.29% (Table 1). The completeness of the assembly was evaluated against the plant-specific Benchmarking Universal Single-Copy Orthologs (BUSCO) database. The completeness was found to be 99.01% indicating robust genome integrity (Table S4). Hi-C data were employed to correct contigs, which were then scaffolded into eight pseudo-chromosomes (Fig. 1C and D), anchoring a total of 290.58 Mb to the chromosomes, accounting for 99.30% of the assembled genome (Table 1).

**Table 1:** Comparison of genomic features of *M. rubra* genome assemblies

Genome features	Zaojia Version 2.0	Zaojia Version 1.0 [8]	Y2012–145 [4]
Length of the total genome (Mb)	292.60	289.92	313.00
Number of contigs	47	1431	511
Contig N50 length (Mb)	36.50	2.16	35.74
Length of the chromosomes (Mb)	290.58	-	279.68
Total size of unanchored contigs (Mb)	2.02	-	33.32
Percentage of assembly in chromosome (%)	99.30	-	89.35
Number of base chromosomes	8	-	8
Number of gap-free chromosomes	8	-	-
Number of gaps	0	7957	2914
Number of telomeres	15	-	-
Number of centromeres	8	-	-
TE size (%)	38.58	36.01	33.05
GC content (%)	37.29	36.80	37.50
Genome BUSCOs (%)	99.01	98.50	94.24
LTR assembly index score	13.21	11.34	6.54
Gene number	28 345	26 325	29 351

The analysis predicted eight centromeric regions and identified 15 telomeres using the seven-base telomeric repeat (CCCTAAA/TTTAGGG), though one telomere of Chr8 had incomplete assembly (Fig. S1). Tables S5 andS6 detail the centromeric and telomeric regions, respectively. Moreover, the assembly produced eight gap-free chromosomes, with the long terminal repeat (LTR) Assembly Index (LAI) of 13.21 (Table 1, Fig. S2), leading to the designation of Zaojia Version 2.0 as a chromosomal-level, Telomere-to-Telomere (T2T), and gap-free reference genome.

Predicted totals of 28 345 genes and 33 502 mRNAs were annotated, with functional annotations for 87.09% and 88.58%, respectively, based on existing databases (Table S7). Repeat sequences, primarily comprising tandem repeats, interspersed repeats, and transposable elements (TEs), constituted 43.43% of the Zaojia Version 2.0 sequences. The constructed repeat database facilitated the identification of 112.89 Mb of TE sequences, representing 38.58% of the repeated DNA (Table S8). Moreover, 164 639 simple sequence repeats (SSRs) were annotated, including a detailed count for single to hexanucleotide SSR sites (Table S9). These data represent a substantial genetic resource for future functional genomics and molecular breeding endeavors in *M. rubra*.

Comparative assessments with the Zaojia Version 1.0 [8] and ‘Y2012–145’ [4] genomes showed significant enhancements in the Zaojia Version 2.0 assembly, with a contig N50 approximately 17 times that of Version 1.0 and all gaps from previous versions filled. The Version 2.0 genome not only demonstrated consistency with ‘Y2012–145’ but also filled gaps in the centromeric regions (Fig. S3). This latest version of the genome was longer by 2.68 Mb and includes 2020 additional genes compared to Version 1.0. The BUSCO score of 99.01% for Version 2.0 also surpassed that of ‘Y2012–145’ at 94.24% (Table 1).

Divergence timing estimated *M. rubra*’s split from *Juglans regia* at 66 Mya (Fig. S4), consistent with previously reported results [24]. OrthoFinder identified 19 312 orthogroups (OGs) among *M. rubra* and eight other analyzed plant species (Table S10), with significant gene family expansions and contractions. In *M. rubra*, there were 1014 OGs expanding and 4258 OGs contracting (Fig. S4). These rapidly changing OGs were associated with chloroplast development, ripening, and stress resistance functions. (Table S11).

### Population structure analyses

A total of 173 bayberry accessions from 10 provinces (Fig. S5) were sequenced, generating 1744.12 GB of clean data. This averaged about 10.08 GB per sample with an average sequencing depth of 36.54× and a mapping ratio of 96.89% (Table S10). The sequences were aligned to the reference genome Zaojia Version 2.0, resulting in the identification of 6 649 674 single nucleotide polymorphisms (SNPs) with Ts/Tv ratios between 2.16 and 2.35 (Tables S12-S13). Genotypic data quality was evaluated with KASP markers for randomly selected SNPs in 173 accessions, with a sequencing accuracy of 98.05% for the selected SNPs (Table S14), confirming high sequencing accuracy.

**Figure 2:**
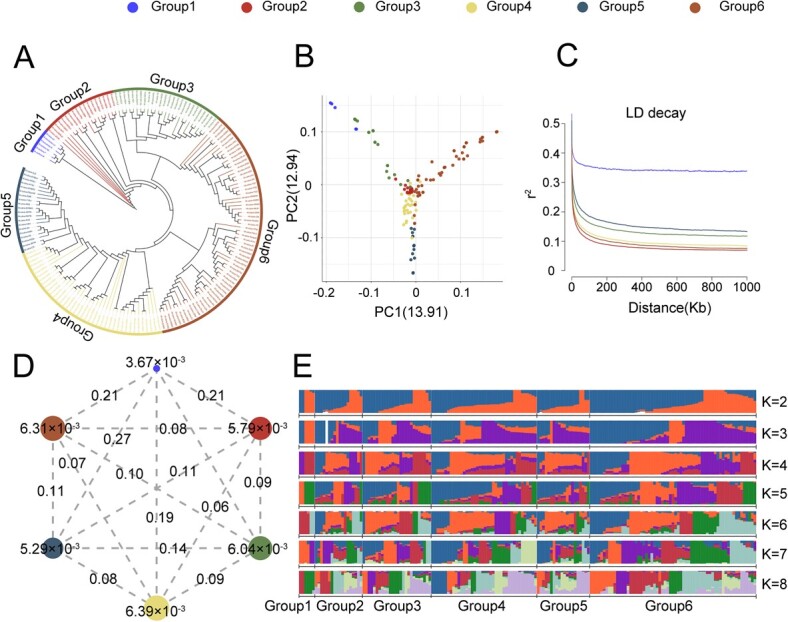
Population Structure and Genomic Diversity in Bayberry. A. NJ phylogenetic tree of 173 bayberry accessions, classified into six groups. Legend colors represent different groups, with Groups 1 to 6 represented as blue, red, green, yellow, grey-blue, and brown, respectively. Legends for Figures B, C, and D correspond accordingly. B. PCA plots categorizing the 173 bayberry accessions into six groups. C. LD decay plots for each group. D. Nucleotide diversity (π) and population divergence (F_ST_) in the six groups, with circle values indicating nucleotide diversity and line values representing pairwise population divergence. E. Genetic makeup of all accessions partitioned into six groups, with model-based clustering from K = 2 to K = 8. Seven distinct colors denote different genetic backgrounds.

Neighbor-joining (NJ) tree, geographic distribution and principal component analysis (PCA) classified the accessions into six groups (Fig. 2A and2B; Table S13). Group 1 was primarily comprised of interspecific germplasm resources, while the other five groups consisted of intraspecific germplasm. Group 1 showed strong genetic divergence from the other five intraspecific groups (pairwise fixation statistic (F_ST_) values ≥0.19, Fig. 2D) and showed the lowest nucleotide diversity (π = 3.67 × 10^−3^, Fig. 2D), consistent with a narrow genetic base. Linkage disequilibrium (LD) decay rates ranked in the following order: Group 2 > Group 6 > Group 4 > Group 3 > Group 5 (Fig. 2C). Each group also exhibited distinctive genetic diversity and phenotypic traits related to fruit quality. Group 2 thus exhibited the fastest LD decay, 18 accessions were primarily from Guizhou and Hunan and exhibited higher levels of genetic diversity (Table S15). The average total soluble solid (TSS) content (12.21%) and the titratable acid (TA) content (1.02%) were the highest in Group 2 (Table S16). In Group 3, 26 germplasms were primarily from Fujian and Guangdong and had the highest levels of flavor-related amino acids, including aspartate (Asp; 116.47 mg/g) and glutamate (Glu; 122.99 mg/g) (Table S16). In Group 4, 40 accessions were primarily distributed across Jiangsu. Group 4 had an average fruit weight (FW) of 12.68 g, which was the highest among the six groups (Table S16), whereas its TSS content was the lowest (9.89%). This suggested a possible negative association between FW and TSS, requiring further investigation. Additionally, Group 4 showed the highest nucleotide diversity (π = 6.39 × 10^−3^, Fig. 2D). In Group 5, 20 germplasms exhibited the lowest levels of nucleotide diversity. These 20 accessions largely consist of recently-bred, manually-selected varieties primarily distributed in northern Zhejiang. The fruits were primarily red and white, with respective average lightness (L*) and red-green (a*) color values of 26.58 and 9.87, which were significantly higher than the other groups (Table S16). Group 6 exhibited the highest heterozygosity and polymorphism, and 63 accessions in this group were primarily distributed across central and southern Zhejiang. The respective average total sugar (TS) and acid-sugar ratio (AS) in Group 6 were 88.00 mg/g and 11.20, respectively (Table S16). The high AS was consistent with the superior sweet/sour flavor of fruits from Group 6.

We next investigated the population genetic structure for clusters (K) from 2 to 8 based on 6.65 million SNPs among the 173 bayberry accessions. The optimal K value of 7 was determined using the chooseK.py script in fastStructure (Fig. 2E). Group 1 mainly consisted of six interspecific germplasms, and the genetic background of the population was relatively single, mainly included two genetic backgrounds, corresponding to two different origins of germplasm resources. The genetic background of other intra-species germplasm groups was relatively rich, from Group 2 to Group 6 all shared seven different genetic backgrounds, similar with previous studies [25].

The F_ST_ analysis, comparing the top 1% significantly different loci and related genes between Group 5 and Groups 2, 3, 4, and 6. Windowed F_ST_ values across the whole genome further revealed broad genomic divergences between the groups. Annotation showed that major functional genes differing significantly between Group 5 and Groups 2, 3, 4, and 6 included MYB, WD40, and sugar transporter genes (Fig. S6). Furthermore, functional genes that differed significantly between Groups 5 and 3 were found to represent mainly the Glu/Leu/Phe/Val dehydrogenase family and the glutamate receptor (Fig. S6B), while genes differing between Groups 5 and 4 mainly included genes of the Cytochrome P450 and MAP families (Fig. S6C), demonstrated that genetic divergence changed the fruit quality.

### Phenotypic data analysis

Fruit quality, including both internal and external characteristics, is a key determinant of the commodity value of Chinese bayberries. Fruit appearance includes factors such as color, size, and shape, while internal qualities include sugar levels and amino acid contents. A total of 29 morphological traits across 136 accessions were evaluated (Table S16). The traits displayed considerable variation (CV > 10%), except for the fruit shape index (FI), indicating significant phenotypic diversity within the germplasm. Most indicators associated with fruit size, color, quality, and amino acid content were positively correlated. Fruit size traits were negatively correlated with fruit sugar and acid content and amino acid content, whereas amino acid content was positively correlated with color- and quality-associated traits. Correlations between fruit size, color, quality, and amino acid content offered insights into trait relationships. All traits were found to fit a normal distribution, verifying their suitability for GWAS (Fig. S7 and Table S16).

### GWAS analyses of bayberry fruit-related traits

Using EMMAX, a GWAS analyzed 29 traits against 6 649 674 SNPs, yielding 1937 significant SNP signals (*P* < 10^−5^) associated with 28 traits (Table S17). Among the traits, L* values were associated with the greatest number of SNPs (n = 580), followed by glutamic acid (Glu) (n = 272) and arginine (Arg) (n = 164). Additionally, 961 SNPs were associated with fruit appearance quality traits (fruit size and color traits), with 39.44% (379/961) and 26.18% (250/961) located in Chr6 and Chr4, respectively. There were 976 SNPs related to intrinsic quality traits (sugar and acid-related traits and amino acids content), of which 50.31% (491/976) were located in Chr3. These 1937 associated SNPs were located within loci corresponding to 1039 genes. The distribution of SNPs across traits and chromosomes revealed insights into the genetic determinants of fruit quality, with subsequent expression analyses of genes linked to extreme phenotype transcriptomes to verify differentially expressed genes (DEGs) shedding light on the genetic regulation of fruit quality in the Chinese bayberry.

### Fruit color-associated SNPs

Overall, 783 SNPs and 441 genes related to fruit color were identified. Notably, 239 (30.52%) and 279 (35.63%) of these SNPs were located on Chr4 and Chr6, respectively. L* values were associated with 580 SNPs and 323 genes, while a* values corresponded to 40 SNPs and 38 genes, and yellow-blue (b*) values were related to 163 SNPs and 80 genes (Table S17). Transcriptomic sequencing of mature fruits from the representative SJZ and ZJ germplasms verified the gene associations (Fig. S8).

**Figure 3:**
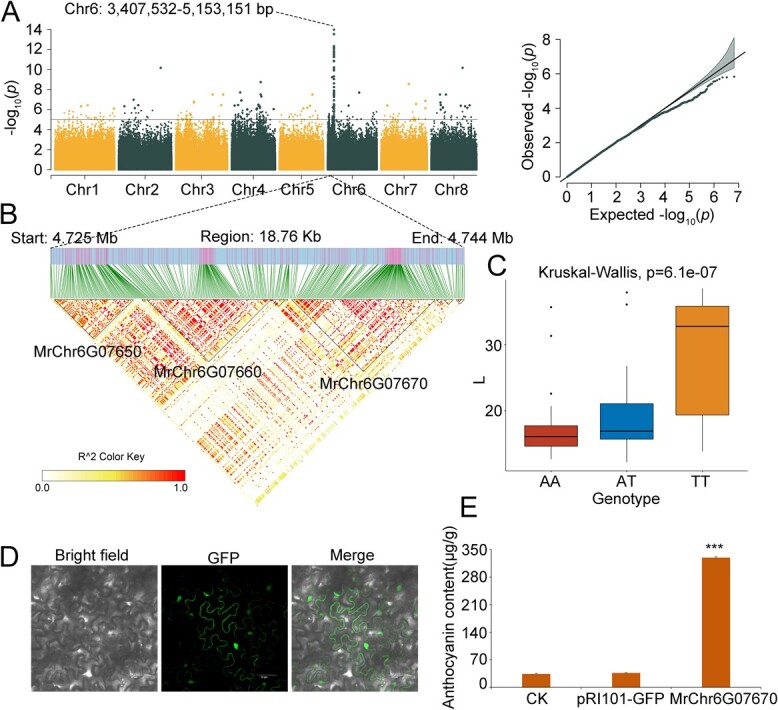
GWAS of Fruit Color Traits Associated with SNPs. A. Manhattan plot showing negative log_10_*p*-values from the EMMAX GWAS scan for L* across the eight chromosomes. A notable SNP cluster spans Chr6: 3 407 532-5 153 151 bp. B. LD block (18.76 kb) displaying the relationships among *MrChr6G07650*, *MrChr6G07660*, and *MrChr6G07670*, with color intensity indicating the degree of LD. C. Haplotype analysis of the SNP at Chr6: 4741659 within the exon of *MrChr6G07670*, showing the influence of AA (red), AT (blue), and TT (yellow) genotypes on the L* value. The x-axis represents genotypes, and the y-axis denotes L* values. D. Subcellular localization of *MrChr6G07670* shown by transformation of tobacco leaves with the overexpression vector *pRI101-GFP-MrChr6G07670*, indicating localization in the cytosol, nucleus, and cell membrane. E. Comparative anthocyanin levels in control (CK) leaves, and leaves transformed with the empty vector (*pRI101-GFP*) and *MrChr6G07670*-overexpression vector. Anthocyanin content (μg/g) is represented on the x-axis, *** indicating a significant difference at p < 0.01.

A significant SNP cluster on Chr6, ranging from 3 407 532 to 5 153 151 bp, was found in proximity to two adjacent genes, *MrChr6G07650* and *MrChr6G07660*, coding for MYB transcription factors (Fig. 3A and B), previously identified as color-associated genes [19, 20], are known regulators of anthocyanin biosynthesis in Chinese bayberry fruits. *MrChr6G07650* was particularly linked to the L*, a*, and b* co-associated SNPs at Chr6: 4728368 while *MrChr6G07660* had L* and b* associated SNPs at Chr6: 4737042. The upregulation of these genes in SJZ and ZJ, especially in SJZ, correlated with the L* and b* values inversely with fruit color. Our findings align with previous research [19, 20], verifying the accuracy of GWAS SNP identification in this study.

Furthermore, a gene cluster exhibited a missense variant at Chr6: 4741659_A > T within an exon of *MrChr6G07670*, adjacent and closely linked to *MrChr6G07650* and *MrChr6G07660* (Fig. 3B). This variant resulted in a tyrosine to phenylalanine substitution at position 39. Haplotype analysis linked this variant to increased L* values, with the homozygous genotype having the most significant effect (Fig. 3C). In tobacco leaves transiently transformed with a *MrChr6G07670* overexpression vector (*pRI101-GFP-MrChr6G07670*), GFP expression was observed in the cytosol, nucleus, and cell membrane (Fig. 3D). The anthocyanin levels in these leaves were approximately ten times higher than those in the controls (Fig. 3E). This result demonstrated that this SNP cluster comprising *MrChr6G07650*, *MrChr6G07660*, *MrChr6G07670*, and other genes was a QTL, collectively determining the color of the bayberry fruit.

### Fruit size-associated SNPs

**Figure 4:**
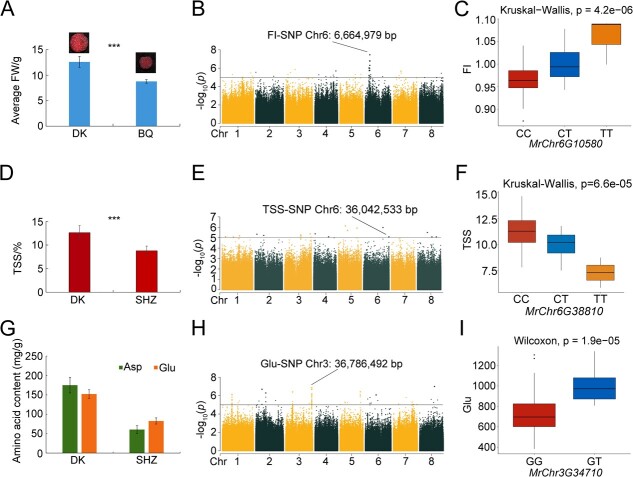
Analysis of SNPs Linked to Fruit Size, Sugar, Acid, and Amino Acid Contents. A. Differences in fruit size between DK (large fruit) and BQ (small fruit), demonstrating a significant size difference (*** p < 0.01, the same below in Figures D and E). B. Manhattan plot for FI from GWAS, with negative log_10_ p-values plotted against chromosomal positions. The SNP at Chr6:6 664 979 bp marks the peak location. C. Haplotype analysis for the SNP at Chr6:6 664 979 in the exon of *MrChr6G10580*, showing the impact of CC (red), CT (blue), and TT (yellow) genotypes on FI. The x-axis indicates genotypes; the y-axis represents FI values. D. TSS variability between DK (higher TSS) and SHZ (lower TSS). E. Manhattan plot for TSS from GWAS, with the significant SNP at Chr6:36 042 533 bp highlighted. F. Haplotype analysis of the SNP at Chr6:36 042 533 within the exon of *MrChr6G38810*, showing the effect of CC (red), CT (blue), and TT (yellow) genotypes on TSS. The x-axis represents genotypes; the y-axis indicates TSS values. G. Differences in amino acid contents between DK (high in Glu and Asp) and SHZ (low in Glu and Asp). H. Manhattan plot for Glu from GWAS, with the peak SNP at Chr3: 36 786 492 bp indicated. I. Haplotype analysis for the SNP at Chr3:36 786 492 bp within the exon of *MrChr3G34710*, detailing the influence of GG (red) and GT (blue) genotypes on Glu levels. The x-axis denotes genotypes; the y-axis signifies Glu values.

In total, 178 SNPs and 92 genes were significantly linked to bayberry fruit size. These associations were primarily with FI (n = 120), followed by FW (n = 29), fruit broadwise diameters (BD) (n = 15), and lengthwise diameter (LDI) (n = 14). A concentration of 100 SNPs on Chr6, particularly between 4 518 777 to 7 970 479 bp, was found to correspond significantly to 12 genes (Table S17). Transcriptomic sequencing of mature fruits from small Biqizhong (BQ, average FW = 8.90 g) and large Dongkui (DK, average FW = 20.15 g) germplasms verified these associations (Fig. 4A).

The FW-associated gene *MrChr6G08980*, located at 5 666 736 bp on Chr6, is an AP2/ERF transcription factor implicated in the regulation of hormones such as ethylene, cytokinin, and auxin, affecting plant development and fruit morphogenesis [26]. It was differentially expressed in BQ and DK (Fig. S9), highlighting its role in bayberry fruit morphogenesis. An FI-associated SNP at 6 664 979 bp (C > T) on Chr6 linked to *MrChr6G10580*, identified as an α/β hydrolase family member, is suggested to interact with gibberellin-activated pathways that may regulate fruit development [27] (Fig. 4B). Haplotype analysis suggested that this SNP was associated with increased FI, with the homozygous variant genotype having the most significant impact (Fig. 4C). Additionally, an LDI-associated SNP at 7 470 654 bp on Chr6 corresponded to *MrChr6G11710* (MAP), a putative regulator of fruit elongation through microtubule stabilization [28]. These findings propose potential roles for these genes as regulators of bayberry fruit size and development and as candidate genes for breeding programs.

### Fruit sugar and acid-associated SNPs

Overall, 78 SNPs and 71 genes were found to be significantly associated with fruit sugar and acid traits. The majority of the SNPs were related to TSS (n = 35), followed by TA (n = 19), TS (n = 10), Vitamin C (Vc) (n = 9), and AS (n = 5), with 25 SNPs (32.05%) located on Chr3 (Table S17). TSS, indicative of a range of compounds in fruit juices, was explored through transcriptomic sequencing of mature fruits from SHZ (average TSS = 8.78%) and DK (average TSS = 12.61%) germplasms (Fig. 4D).

A group of 35 TSS-related loci were identified, with 20 SNPs situated on Chr3 (23 070 886 to 37 758 513 bp). *MrChr3G36470*, a sorting nexin family gene, associated with TSS and upregulated in DK and SHZ (Fig. S10), plays a role in early fruit and endocarp tissue development [29]. *MrChr6G38810* (SNP at Chr6:36042533 bp) was significantly associated with TSS, an LRR-RLK family gene, played an important role in fruit development (Fig. 4E) [30]. Haplotype analysis revealed that SNP variants at this site decreased TSS, with homozygous contributing the most (Fig. 4F). Downregulation of *MrChr1G06130* (SNP at Chr 1: 5305139 bp), associated with TSS, pointed to its role as an SBP family gene influencing grain size, shape, and fruit quality [31, 32]. A SNP at 8577962 bp on Chr 2 linked to *MrChr2G09190*, an upregulated PPR family gene, affects fruit color and taste by modulating carotenoid and chlorophyll pigment accumulation [33].

### Amino acid-associated SNPs

Of the 17 amino acids assessed, 16 had significant associations with 898 SNP loci and 435 genes. Glu had the highest number of associated SNPs (272), followed by Arg (164) and Asp (160), with 466 SNPs (51.89%) on Chr3 (Table S17). Genes influencing Asp and Glu levels may provide opportunities to enhance bayberry taste and nutritional value. Asp (42.50–236.41 mg/g) and Glu (56.42–196.97 mg/g) were the most abundant flavor-related amino acids in bayberry fruits (Table S14). Transcriptomic sequencing from SHZ (average Asp and Glu: 60.63 and 82.66 mg/g) and DK (average Asp and Glu: 175.03 and 152.24 mg/g) germplasms, with distinct Asp and Glu content, verified the associated genes (Fig. 4G).

A Chr3 SNP at 36 786 492 bp linked to *MrChr3G34710* (Fig. 4H), annotated as an L-type lectin-domain-containing receptor kinase and involved in fruit pulp quality, was downregulated in SHZ and DK (Fig. S11). Haplotype analysis associated this variant with higher Glu content, with heterozygotes (GT > GG) affecting Glu to the greater extent (Fig. 4I). An Asp and Phe-related SNP at Chr8 (29 043 544 bp) corresponded to *MrChr8G28780*, an AP2 transcription factor downregulated in SHZ and DK. *MrChr3G09670*, associated with Asp and located at Chr3 (9 595 256 bp), was identified as an AP2 transcription factor involved in ripening, affecting color, texture, and flavor [34]. A Chr6 SNP (17 757 960 bp) related to Glu and tyrosine (Tyr) was associated with *MrChr6G24160*, a bHLH transcription factor that regulates metabolite biosynthesis and was upregulated in SHZ and DK [35]. Additionally, *MrChr3G09340* (annotated as WD40), linked to an Asp-associated SNP on Chr3 (9 220 224 bp), affects grain development and metabolite accumulation [36, 37].

### Co-expression network and pathway analysis

**Figure 5:**
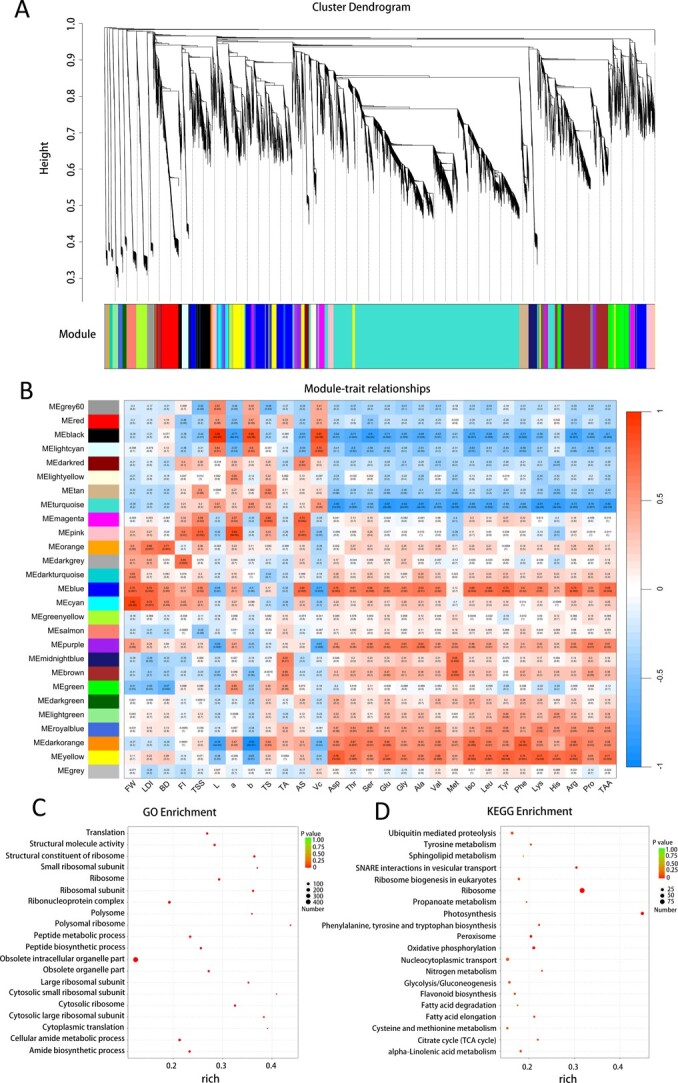
Clustering of Module Eigengenes and Correlations between the Gene Module and 29 Fruit-Quality Traits. A. cluster dendrogram showing 27 gene co-expression modules was built based on the dissimilarity of the topological overlap. B. Heat map of correlation between 29 traits and expressed genes. The correlation coefficient in each cell represents the correlation between the gene module and fruit-quality traits, decreasing in size from red to blue. The corresponding p-value is also annotated. C. GO enrichment analysis. D. KEGG pathway analysis. The circle size represents the number of genes, and color changes from red to green indicate higher p-values in Figures C and D.

To gain further insights into the relationship between fruit quality traits and gene expression in bayberry, a weighted gene correlation network analysis (WGCNA) using 23 470 genes was conducted. A total of 27 co-expression modules were identified based on similar expression patterns (Fig. 5A). The Turquoise module contains the most genes (8992 genes), followed by blue module (2466 genes). The blue module had the highest association with fruit quality in the heat map of the module-trait relationship, and this module was selected as the regulatory relationship between fruit quality traits and gene expression for further analysis (Fig. 5B). Totally, 20 fruit quality traits (15 amino acid traits and FW, LDI, BD, AS, TSS) showed a high correlation with the blue module. According to Gene Ontology (GO) and Kyoto Encyclopedia of Gene and Genome (KEGG) pathway enrichment analyses of genes in blue module. GO pathway analysis indicated that these genes were mainly enriched in the peptide biosynthetic and metabolic process, and amide biosynthetic and metabolic process (Fig. 5C). KEGG pathway analysis indicated significant enrichment in pathways associated with phenylalanine, tyrosine, and tryptophan biosynthesis, tyrosine metabolism, and flavonoid biosynthesis (Fig. 5D). These results indicated that the genes in the blue module were mainly involved in the regulation of amino acid and flavonoid biosynthesis and metabolism, and jointly participated in the fruit size, AS and TSS formation process, thus influencing fruit quality.

## Discussion

At the chromosomal level, a T2T gap-free reference genome can enhance the identification of structural variants and facilitate an integrated assessment of genotype–phenotype relationships. It provides insights into mechanisms and genomic evolution while elucidating the genetic underpinnings of specific traits. The T2T genome data reported for maize, grapevine, and other plant species have laid the methodological foundation for assembling the bayberry genome [38, 39]. In this study, we initially assembled the genome using Hifiasm, removed redundant sequences, applied Hi-C data for chromosome scaffolding, and refined it with Juicerbox, which resulted in seven retained gaps. These were addressed using HiFi data, culminating in the gap-free Zaojia Version 2.0 chromosome genome, representing a comprehensive assembly and patching process that ensures a high degree of genome continuity and integrity. When compared to two previously published *M. rubra* genome sequences, Zaojia (Version 1.0) [8], and ‘Y2012–145’ [4], which were primarily scaffold assemblies with suboptimal sequence continuity, Zaojia Version 2.0 exhibited superior sequence continuity and integrity (Fig. S3 and Table 1). These advances underscore the high quality of our *M. rubra* genome assembly, positioning it as a valuable resource for studying the evolution and domestication of Chinese bayberry and for uncovering the genetic basis of bayberry fruit quality.

Selective sweep analysis conducted on 173 bayberry germplasms corroborated earlier studies that investigated the origins and evolution of Chinese bayberry plants [11, 25]. The germplasms in our study clustered into six groups, with seven different genetic backgrounds were shared among five intraspecific germplasm groups, indicative of shared origins or genetic backgrounds. Previous research has suggested that *M. rubra* originated from the Hengduan Mountains in China [11]. In our study, germplasms in Group 2 were primarily distributed in the Guizhou and Yunnan provinces, near the Hengduan Mountains. Group 2 germplasms exhibited the lowest degree of artificial selection and fastest LD decay, suggesting a lower degree of domestication, providing further verification of the conclusions on the origin and consistent with earlier findings. The combination of the LD decay rates of Groups 2 to 6, analysis of the population genetic structure, and speculation on the geographic locations of the groups suggested a west-to-east domestication trajectory for the Chinese bayberry germplasm resources, with Zhejiang emerging as a central domestication hub. This rich genetic diversity, particularly in Groups 2 and 4, presents opportunities for hybridization to enhance genetic variability and provides genetic resources for targeted trait improvement in TSS and FW, thus laying the groundwork for advancing *M. rubra* cultivation and establishing a robust core germplasm resource bank. Additionally, the ZJ genomic variety included genetic information from both BQ and DK, indicative of a hybridization event the development of the ZJ variety, and possessed both the early ripening characteristic of BQ and the large fruit of DK. According to the F_ST_ analysis, the main genes showing significantly different loci between the groups were MYB, WD40, the sugar transporter, the Glu/Leu/Phe/Val dehydrogenase family, the glutamate acceptor, and the cytochrome P450 and MAP families. These genes have previously been shown to play key roles in biological processes such as fruit coloration, the transport and accumulation of nutrients such as sugars and amino acids, and fruit development [19, 28, 40–42]. The differences in these loci and genes in different groups should be the main reasons for the domestication of traits such as fruit color, taste, amino acid content, and size.

The large-scale GWAS analysis of *Myrica* L. species, resulting in the identification of 1937 SNP signals, illuminated critical genomic sites associated with functional bayberry genes. A significant proportion of SNP-associated sites (65.62%) linked to fruit appearance quality (traits such as size and color) are concentrated in Chr4 and Chr6, while 50.31% of those related to internal quality (sugar, acid, and amino acid content) predominantly localize to Chr3. These specific genomic regions serve as valuable reference points for enhancing bayberry quality. In the fruit color association analysis, two previously reported MYB genes were identified, affirming the role of the MYB-bHLH-WD40 transcriptional complex in the regulation of anthocyanin biosynthesis in bayberry fruit [19, 21]. Additionally, a newly characterized MLP-like gene (*MrChr6G07670*) adjacent to the MYB genes (*MrChr6G07650* and *MrChr6G07660*) was found to significantly influence both anthocyanin synthesis and fruit color formation. The QTL on Chr6: 3407532-5 153 151 bp, which includes the MYB genes, MLP-like protein, and others, collectively regulate bayberry fruit color. Furthermore, our GWAS revealed functional genes related to fruit size, such as AP2/ERF transcription factors and the MAP protein, known to regulate plant development and influence fruit morphology [43, 44]. The microtubule-associated protein MAP interacts with IQD, impacting fruit shape formation [28]. Genes related to sugar and acid content, including SBP and PPR gene families, were also identified, implicating them in the development of various tissues and the regulation of plant growth [31–33]. These loci and genes represent crucial reference points for studying bayberry fruit size, shape, quality, and taste formation. They could be utilized for developing specific functional markers for molecular-assisted breeding. However, further research will be essential to validate these findings and explore interactions in greater detail.

Amino acids were the important component of the fruit quality of Bayberry, and amino acids synthesis and metabolism were also important pathways for regulating fruit development and other quality formation. For instance, amino acids such as phenylalanine, which participate in anthocyanin synthesis through the phenylpropanoid pathway, play a crucial role in the development of fruit color and quality [45]. In the association analysis with amino acids, important genes related to fruit development and quality formation, such as AP2, bHLH, and WD40, were also discovered, such as AP2, bHLH, and WD40, which have been implicated in the development and quality of fruit in various species [26, 35–37]. Additionally, the AP2 gene can regulate the MYB gene, affecting flavonoid synthesis [46]. Therefore, we speculated that genes related to the synthesis and metabolism of amino acids also play an important role in the formation of bayberry quality, which was also reflected in WGCNA analysis. According to the WGCNA that can analyze correlations between gene expression and quality-associated fruit traits, 15 amino acids and FW, LDI, BD, AS, and TSS were found to be strongly correlated with the blue module. GO and KEGG enrichment analyses showed that blue module genes were enriched in pathways associated with amino acid and flavonoid biosynthesis and metabolism. This suggests a similar mechanism underlying the accumulation of these amino acids and fruit size, AS, and TSS traits. Co-expressed genes involved in pathways such as amino acid synthesis metabolism and flavonoid synthesis metabolism jointly regulate amino acid accumulation and fruit size, AS, and TSS traits formation, as well as further verifying the association between amino acids and fruit quality. This provides a foundation that further reinforces the connection between fruit-quality traits and gene expression in bayberry, establishing a basis for the construction of gene regulatory networks.

In conclusion, the completion of the T2T genome and identification of excellent allelic variations in *M. rubra* pave the way for exploring and improving traits such as color, size, the sugar-to-acid ratio, and amino acid levels, all of which are attributes of considerable importance in bayberry breeding and production. The findings also clarify the population structure of *M. rubra* germplasm resources, which is anticipated to significantly impact future bayberry breeding, conservation, and utilization.

## Materials and methods

### Materials and Phenotyping

We selected the Zaojia cultivar of *M. rubra* for genomic sequencing, a variety known for early maturity, dwarf stature, and dark purple fruits with a sweet and sour flavor, popular among both producers and consumers. Other germplasms with extreme phenotypes included SJZ white germplasm, DK representing large fruit-type germplasm and high TSS and amino acid contents, SHZ red germplasm with low TSS and amino acid contents, and BQ representative of a small fruit-type germplasm.

A total of 173 bayberry accessions were sequenced, comprising 167 intraspecific *M. rubra* resources and 6 interspecific materials from ten provinces in the primary bayberry production area of China (Fig. S5; Table S10). These accessions encompassed white fruit germplasm (5.99%; 10/167), red fruit germplasm (41.32%; 69/167), and black-purple fruit germplasm (52.69%; 88/167). The fruits varied significantly across accessions concerning their color, size, and other quality-related traits. The six interspecific germplasms represented *M. cerifera* (n = 3), *M. esculenta* (n = 2), and *M. nana* (n = 1) and were collected from the national Chinese Bayberry Germplasm Resources Garden (Suzhou) and the Zhejiang Chinese Bayberry Germplasm Resources Garden (Linhai), China.

Due to variations in maturity and distribution, mature fruit samples were collected in stages, with successful collection from only 136 germplasms. Random fruit samples (1 kg each) were collected from these 136 cultivars, four size-related traits, FW, LDI, BD, and FI. Ten fruits were randomly selected from each per cultivar and weighed with an electronic balance to determine FW: the process was repeated ten times. LDI and BD were measured using electronic digital vernier calipers to calculate FI (LDI/BD). Three color-related traits, L*, a*, and b* values, were assessed. A portable color difference meter (CR-400, Konica Minolta, Japan) was used to evaluate fruit coloration, and L*, a*, and b* values were recorded. Five sugar- and acid-related traits, the TSS, TA, TS, AS, and Vc contents, were assessed. The TSS content was measured with an Abbe refractometer, TA content was determined by the NaOH titration method, and TS content was measured via anthrone colorimetry. AS was defined as follows: total sugar content/titratable acid content. The Vc content was measured spectrophotometrically. For all content testing, fresh samples were weighed. For each germplasm, 20 fruits were randomly selected and ground to a pulp after core removal. These samples were used for measurements of free amino acid content using a Hitachi L-8900 automatic amino acid analyzer. In total, the levels of 16 amino acids were assessed, Asp, threonine (Thr), serine (Ser), Glu, glycine (Gly), alanine (Ala), valine (Val), methionine (Met), isoleucine (Iso), leucine (Leu), Tyr, phenylalanine (Phe), lysine (Lys), histidine (His), Arg, and proline (Pro). TAA content was calculated from the sum of amino acid levels. Statistical analyses of data were performed using SASS and R, and correlation graphs, mean frequency distributions, and phenotypic data were generated in R.

### Genome survey and PacBio long-read sequencing

An initial genome survey was conducted using 30.99 GB of Illumina short-insert-size data, and clean data were processed for K-mer frequency distribution with Jellyfish v2.2.10 [47], analyzed by GenomeScope v2.0 [48]. For PacBio library construction, about 20 Kb SMRTbell libraries were prepared based on the provided directions, which were then sequenced on a PacBio Sequel II platform, generating 19.21 Gb (65.66×) of long-read data (Table S1).

### Hi-C library construction and sequencing

A Hi-C library was prepared from young leaves of Zaojia trees using the DpnII restriction enzyme and sequenced using the Illumina Hiseq X10 platform, producing PE150 reads [49]. Concurrently, cDNA libraries were sequenced on an Illumina Novaseq 6000 platform, providing 41.97 Gb (143×) of total clean data.

### Genome assembly

Bedtools v 2.25.0 was used to transform the full Sequel subread bam files into the fastq format [50], after which hifiasm with default parameters was used to assemble the extracted reads [51]. Redundant sequences were removed with the purge_dups (https://github.com/dfguan/purge_dups). To support pseudo-chromosome construction, the Hi-C library was prepared according to standard procedure. Using YaHS for chromosome mounting based on Hi-C data [52]. Juicerbox was used to manually correct assembly errors [53], and retaining seven gaps after assembly. Then, using TGS-GapCloser to patch gaps using HiFi data [54], and also using the patch function in RagTag to further patch gaps [55], ultimately producing a chromosome genome version without gaps. Collinearity between the Chinese bayberry genome and the previously published ‘Y2012–145’ chromosome sequences [4] were analyzed using the Minimap2 [56]. LTRs were predicted based on the results of LTR_FINDER_Parallel and ltrharvest, and the LAI was computed with LTR_Retriever [57].

### Gene prediction and annotation

The Program to Assemble Spliced Alignments (PASA) pipeline [58] was utilized for gene prediction, integrating transcript evidence with StringTie [59], Trinity [60]. We performed a comparison with the BUSCO (v3.0.2) [61] embryophyta_odb10 database to assess completeness based on the presence of full-length transcripts. Additionally, and BRAKER v3.0.3 [62] with AUGUSTUS models (v3.2.2) [63] was used to make *ab initio* gene predictions. Protein homology was evaluated against proteins from other plants, specifically *Arabidopsis thaliana* TAIR10, *Carica papaya* ASGPBv0.4, *Populus trichocarpa* v3.1, *Vitis vinifera* Genoscope.12X (https//phytozome.jgi.doe.gov/pz/portal.html#), *Brassica juncea* (GCA_001687265.1), *Brassica napus* Darmor-bzh [56], *Brassica rapa* [64], and *Brassica oleracea* [65]. Functional annotations of the genes were determined using BLASTP and InterProScan [66].

### Re-sequencing and variants identification

The integrity of DNA samples from 173 germplasm accessions was assessed using 1% agarose gel electrophoresis, and DNA concentrations were measured using a Qubit® DNA Assay Kit and a Qubit® 3.0 Fluorometer (Invitrogen, USA). DNA samples were sequenced on the Illumina Novaseq 6000 platform. Initial genome resequencing data for the 173 germplasms were filtered to obtain high-quality data. Short sequences were aligned to the Zaojia Version 2.0 reference genome using the Burrows-Wheeler Aligner (BWA) software with the MEM algorithm [67]. The resulting alignment was converted into the BAM format with SAMtools [68], and Picard (v2.5.0) (http://broadinstitute.github.io/picard/) was used to remove repetitive PCR-derived sequences and calculate actual genomic coverage and sequencing depth. HaplotypeCaller from GATK was used to generate a genomic variant call format file (gVCF) for each sample, which was used for joint genotyping of all samples (via GenotypeGVCFs from GATK). Raw SNPs were filtered with ‘QD < 2.0||MQ < 40.0||FS > 60.0||MQRankSum < −12.5|| ReadPosRankSum < −8.0’. To obtain high-quality allele variation, SNPs were filtered using the following criteria: minimum allele frequency (MAF) > 5%, site deletion rate < 30% in the population, and site depth > 2 per sample.

### Transcriptome sequencing and co-expression module analysis

RNA sequencing was performed on mature fruits, including the following germplasms: SJZ, SHZ, ZJ, BQ, and DK. Each germplasm had three sets of duplicate samples. In total, 15 RNA-seq libraries were prepared and sequenced on the Illumina HiSeq 2500 platform (Illumina). We removed low-quality reads and those containing adaptor sequences with an in-house developed script; mapped paired read expression levels were normalized in the transcripts per million (TPM) format. Raw read counts were calculated using the feature counts module in Subred. DEGs were determined using DESeq2 [69] based on count data, and the resulting P values were adjusted using the false discovery rate (FDR) correction. Genes with log2Fold lower than −1 or higher than 1 and adjusted P value lower than 0.05 were regarded as DEGs. The R package pheatmap package was used for heatmap clustering analysis of DEGs.

To identify modules with high gene correlations, co-expression network analysis was performed using the WGCNA v1.72 package in R v4.2.2 [70]. Transcripts were filtered out using the WGCNA goodGenes function. The cutreeDynamic function was utilized to prune the hierarchical clustering dendrogram of genes obtained through hierarchical clustering. Modules with correlation coefficients (r) exceeding 0.75 were subsequently merged. When using the blockwiseModules modules function to construct a gene co-expression network, an unsigned TOMType was selected. The module eigengenes (MEs) were computed using the module Eigengenes function from the R WGCNA software package. Pearson correlation analysis was used to assess the association between module eigengenes and phenotypic traits.

### Orthogroups and phylogeny

Unique and shared OGs were identified in *M. rubra* using Orthofinder [71] and genomic results were compared to *Eriobotrya japonica* [72], *J. regia* [73], *Actinidia chinensis* [74], *Tetracentron sinense* [75], *Oryza sativa* [76], *Citrus sinensis* [77], *Malus domestica* [78], and *Casuarina equisetifolia* [79]. The phylogeny differentiation time of *M. domestica* and *M. rubra* was obtained from TimeTree (http://timetree.org), and the differentiation time of each species was then estimated based on R8s. Finally, the CAFÉ tool [80] was used to calculate gene family contraction and expansion for the nine analyzed species.

### KASP validation

SNPs within 50 bp upstream and downstream of an interest site were identified using the Cereals DB website 2.0 [81] and used for 6 KASP markers design (Table S12). KASP assays were utilized to validate SNP accuracy based on corresponding read sequences and performed as reported previously [23]. Specifically, KASP-SNPs were compared to corresponding SNPs to establish the number of mismatches, and converted KASP-SNPs were validated in a population of 173 individuals. Based on statistical comparisons between KASP-SNPs and validate SNPs, KASP primer accuracy was determined in the R statistical environment.

### Population structure analyses

VCF tools were used to calculate F_ST_, Pi (π), and other parameters from the high-quality SNP variation data [82]. Plink [83] was used to perform LD and PCA analyses. The NJ evolutionary tree was constructed by Plink filtering of SNPs based on the LD, followed by removal of SNPs with R^2^ values greater than 0.2 with any other SNP within a sliding window of 50 SNPs (increasing by 10 SNPs each time). The operating parameters were ‘plink -vcf pop.recode.vcf -allow-extra-chr --indep-pairwise 50 10 0.2’. The homemade script was used to convert vcf to phy format. The neighbor module in Phylip was used to construct an NJ evolutionary tree [84], the bootstrap value was 1000 and the other values were default. The tree was visualized with iTOL (https://itol.embl.de/). Group structures were analyzed with the fastStructure [85].

### GWAS analysis and candidate gene selection

SNP loci classification and filling were performed using the BEAGLE software [86], and GWAS analysis for fruit quality traits was performed with EMMAX [87]. EMMAX was used to calculate the kinship of the GWAS population, and PC1 and PC2 in PCA were selected as covariates for the correlation analysis. Haplotype analysis was performed to confirm whether particular SNPs contributed significantly to a given phenotype. Linkage candidate regions associated with significant loci were identified using the LDBlockShow (https://pubmed.ncbi.nlm.nih.gov/33126247/). Functional annotation of variants was carried out using SnpEff.

### Subcellular localization analyses

The full-length coding region of *MrChr6G07670,* without the stop codon, was amplified and ligated into the *pRI101-GFP* vector using appropriate primers (Table S12) to produce the *pRI101-GFP-MrChr6G07670* overexpression vector. This vector, or an empty control plasmid (*pRI101-GFP*), was then introduced into *Agrobacterium tumefaciens* via electroporation. Tobacco cultivation, *A. tumefaciens* injection, and fluorescence detection were performed as described previously [88]. Anthocyanin levels in the tobacco leaves were measured spectrophotometrically.

## Supplementary Material

Web_Material_uhae033

## Data Availability

The resequencing and transcriptome data presented in the study are deposited in SRA (http://www.ncbi.nlm.nih.gov/bioproject/936999), and accession number is PRJNA936999; the HiFi and Hi-C data presented in the study are deposited in SRA (http://www.ncbi.nlm.nih.gov/bioproject/937074), and accession number is PRJNA 937074. The genome sequences Zaojia Version 2.0 described in this article were submitted and released to http://cotton.zju.edu.cn/source/Myrica_rubra.zip.
