# Supplementary material for: T2T reference genome assembly and genome-wide association study reveal the genetic basis of Chinese bayberry fruit quality
Source: Hortic Res. 2024 Jan 30;11(3):uhae033. doi: 10.1093/hr/uhae033 (PMC10940123; doi:10.1093/hr/uhae033)
Supplement: Web_Material_uhae033 [file web_material_uhae033.zip › Supplementary Figures.docx]

**Supplementary Figures**


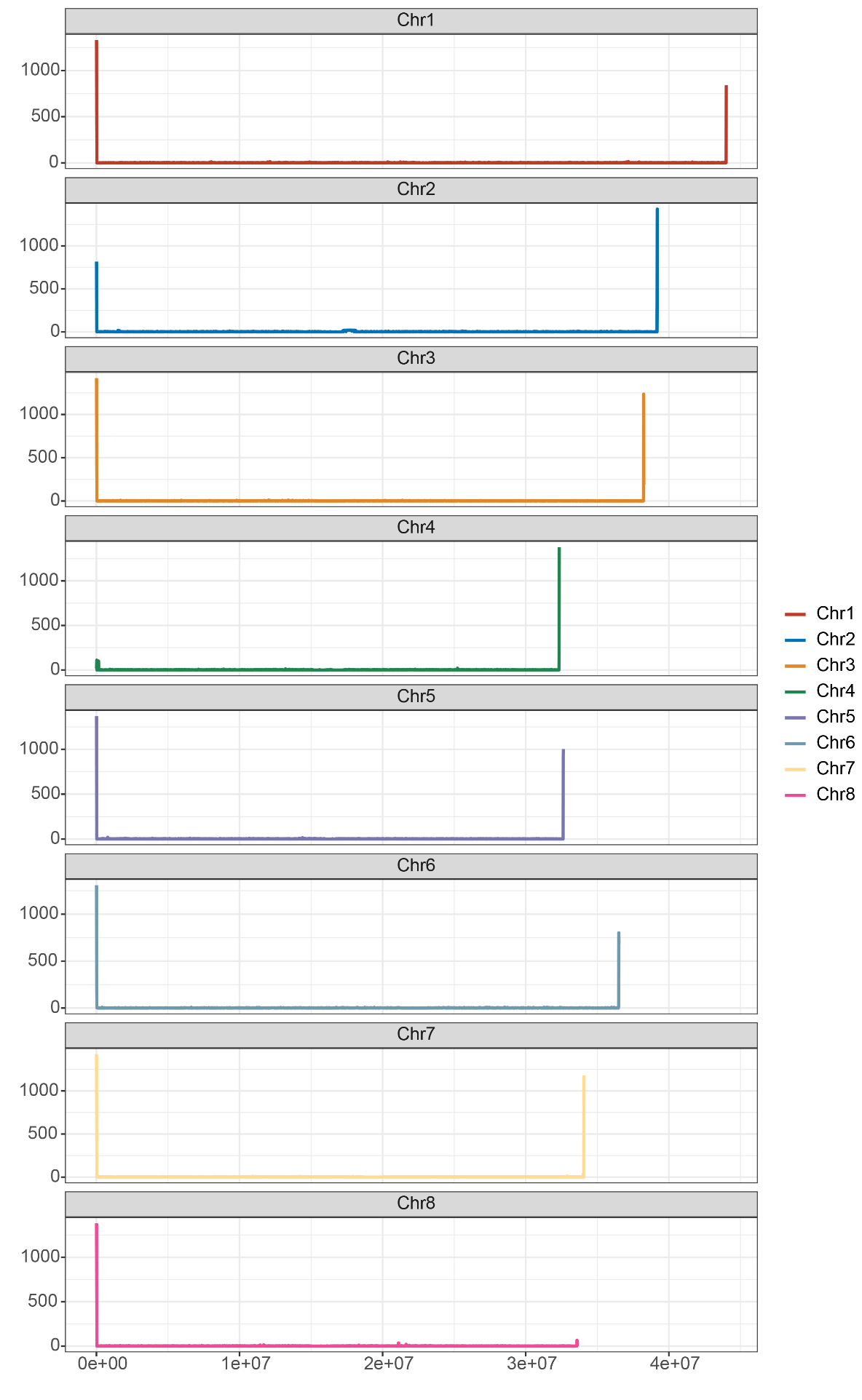


**Figure S1.** Distribution of telomeres on the eight chromosomes.


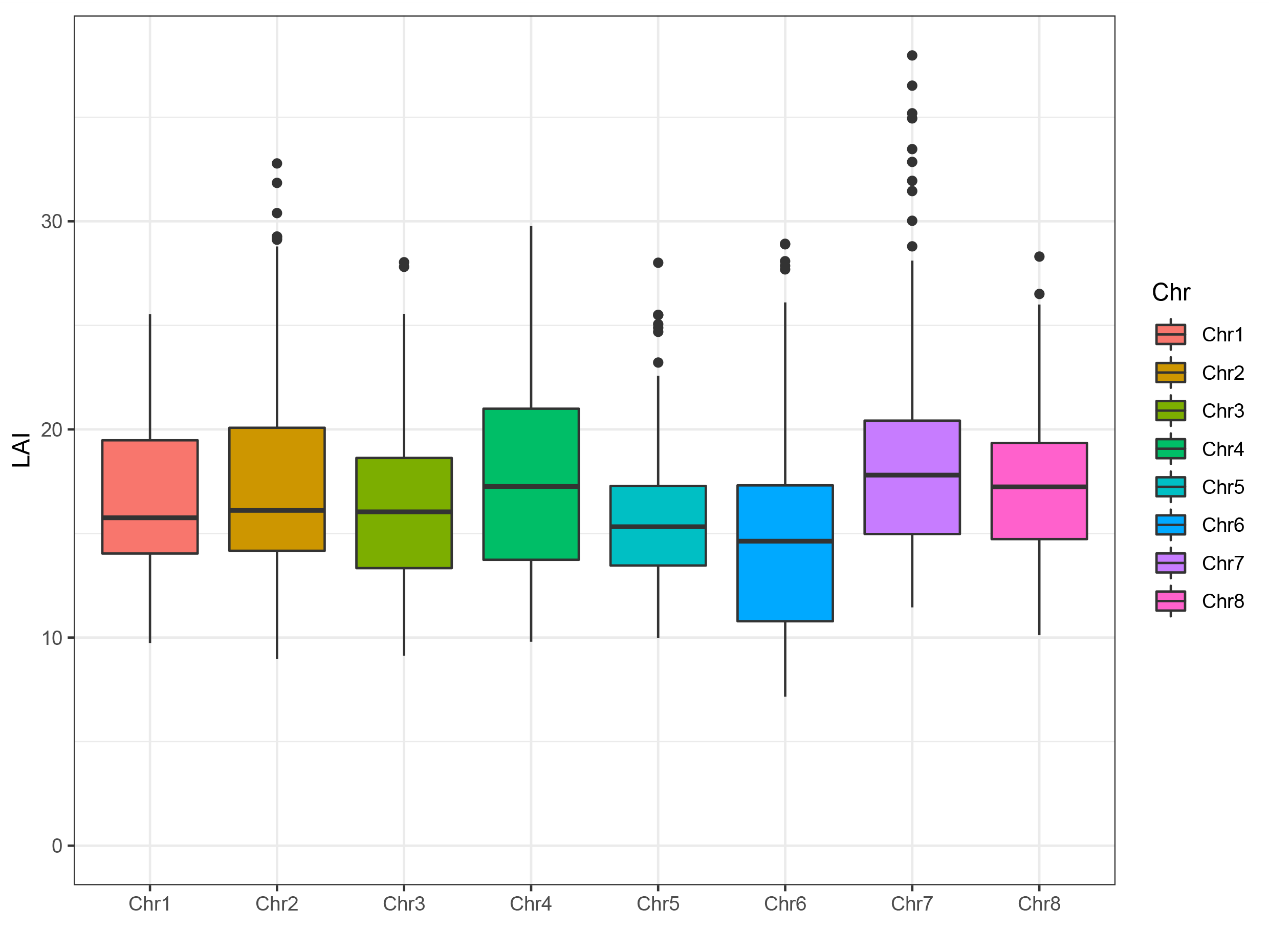


**Figure S2.** The LAI values of the eight pseudo-chromosomes in Zaojia Version 2.


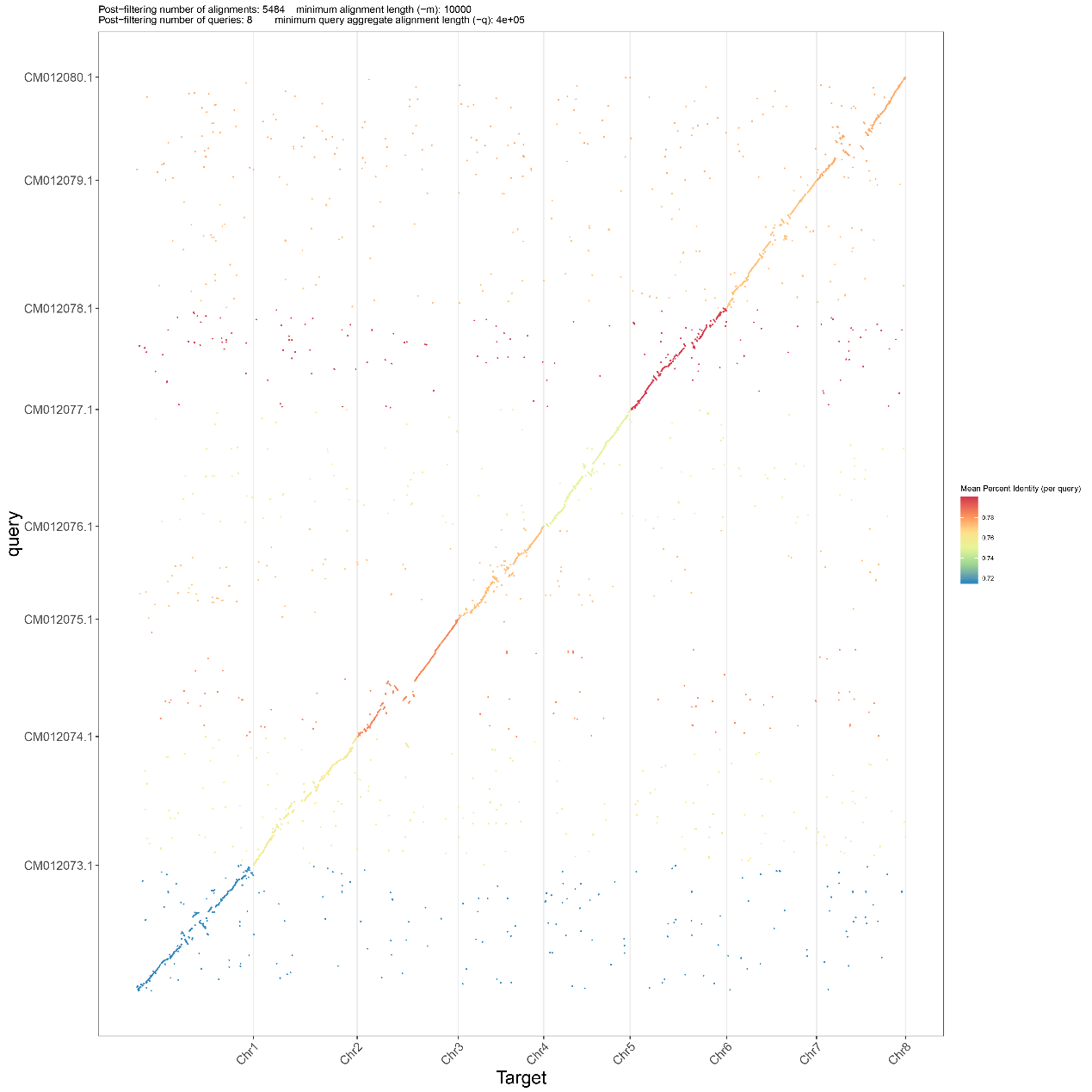


**Figure S3.** Chromosomal sequence consistency between Zaojia Version 2.0 and Y2012-145, from previous research. The query is the Y2012-145 genome version and the target is the Zaojia version 2.0 genome.


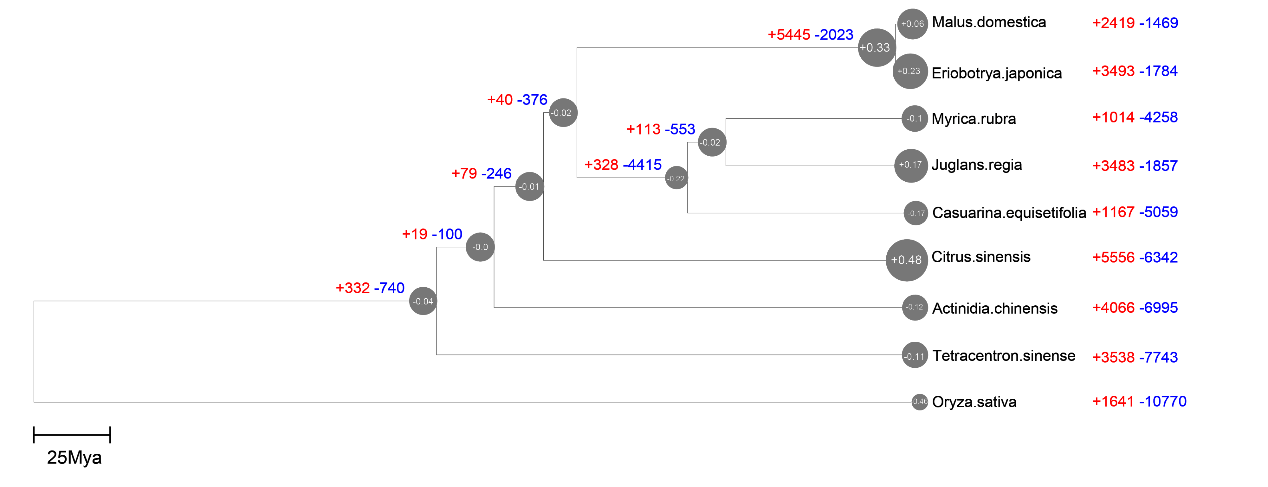


**Figure S4.** Estimates of the times of divergence and evaluation of protein homologs from *M. rubra* and eight other plant species. Scale bar = 25 Mya; red indicates OGs showing expansion and blue indicates OGs showing contraction.


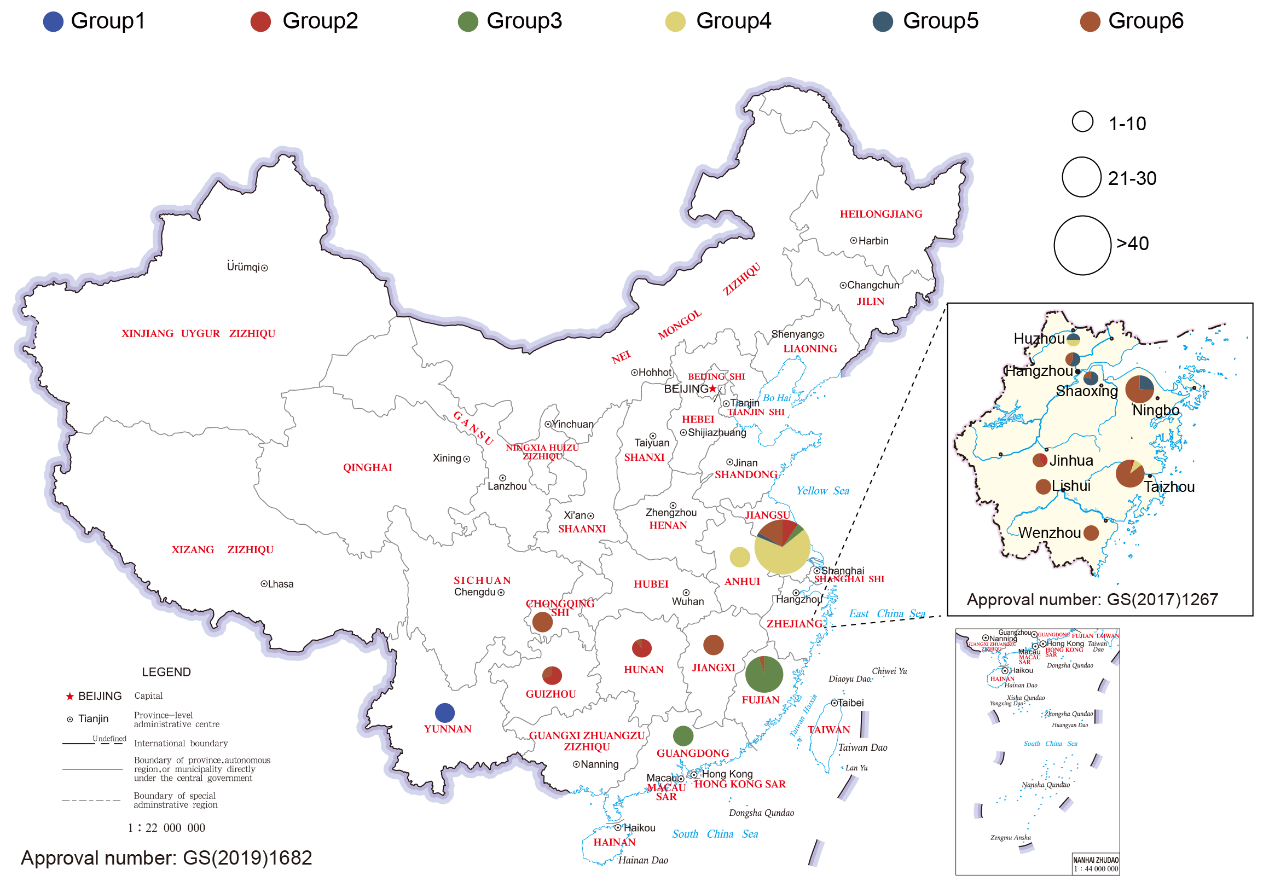


**Figure S5.** Geographic distribution of bayberry germplasm resources, with circle sizes proportional to the number of resources. Legend colors represent different groups, with Groups 1 to 6 represented as blue, red, green, yellow, grey-blue, and brown, respectively. The Standard Map approval number were GS(2019)1682 and GS(2017)1267.


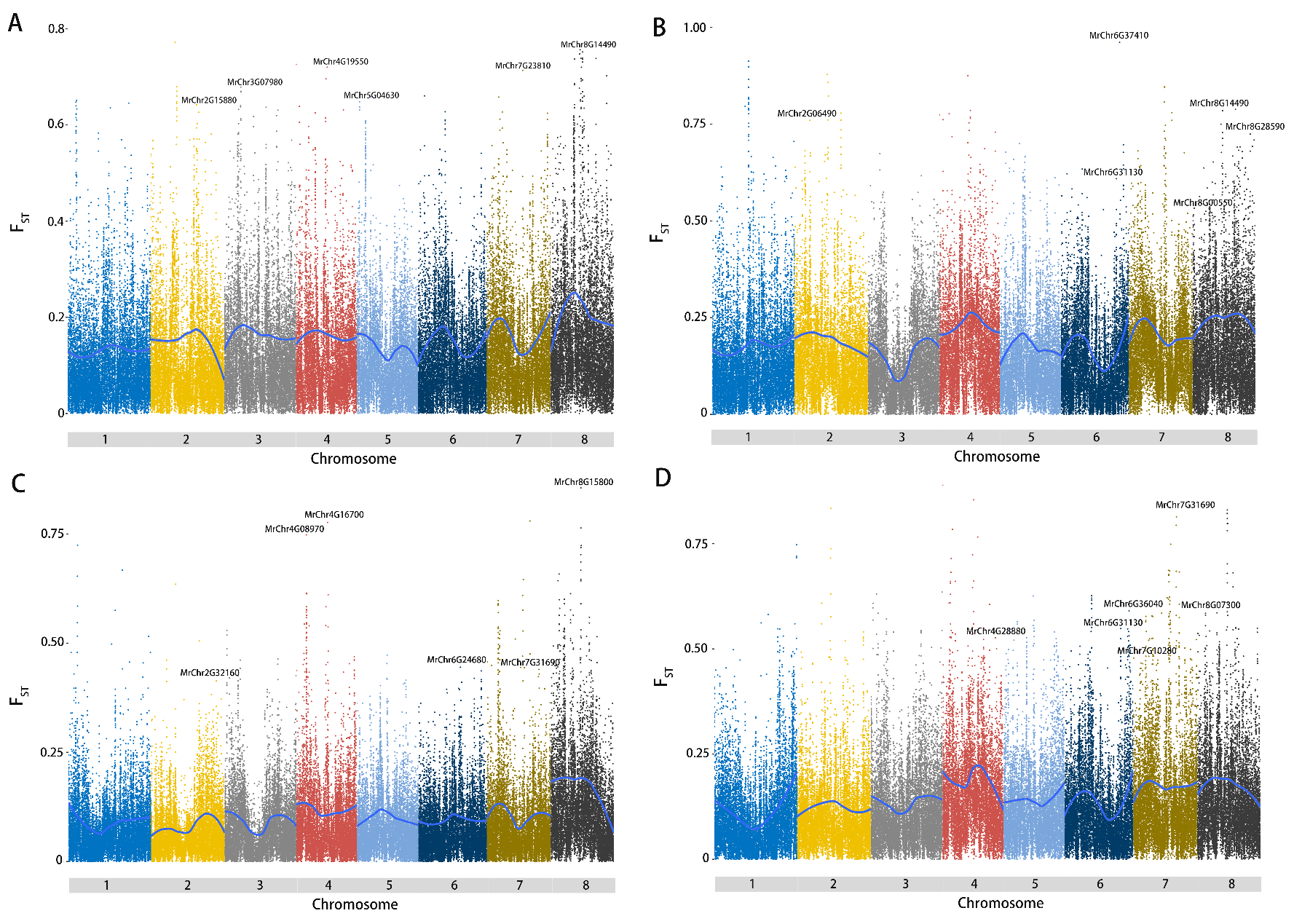


**Figure S6.** Population genetics differentiation (FST) in intraspecific germplasm groups. A. Loci and genes showing significant differences between Groups 5 and 2. B. Loci and genes showing significant differences between Groups 5 and 3. C. Loci and genes showing significant differences between Groups 5 and 4. D. Loci and genes showing significant differences between Groups 5 and 6.


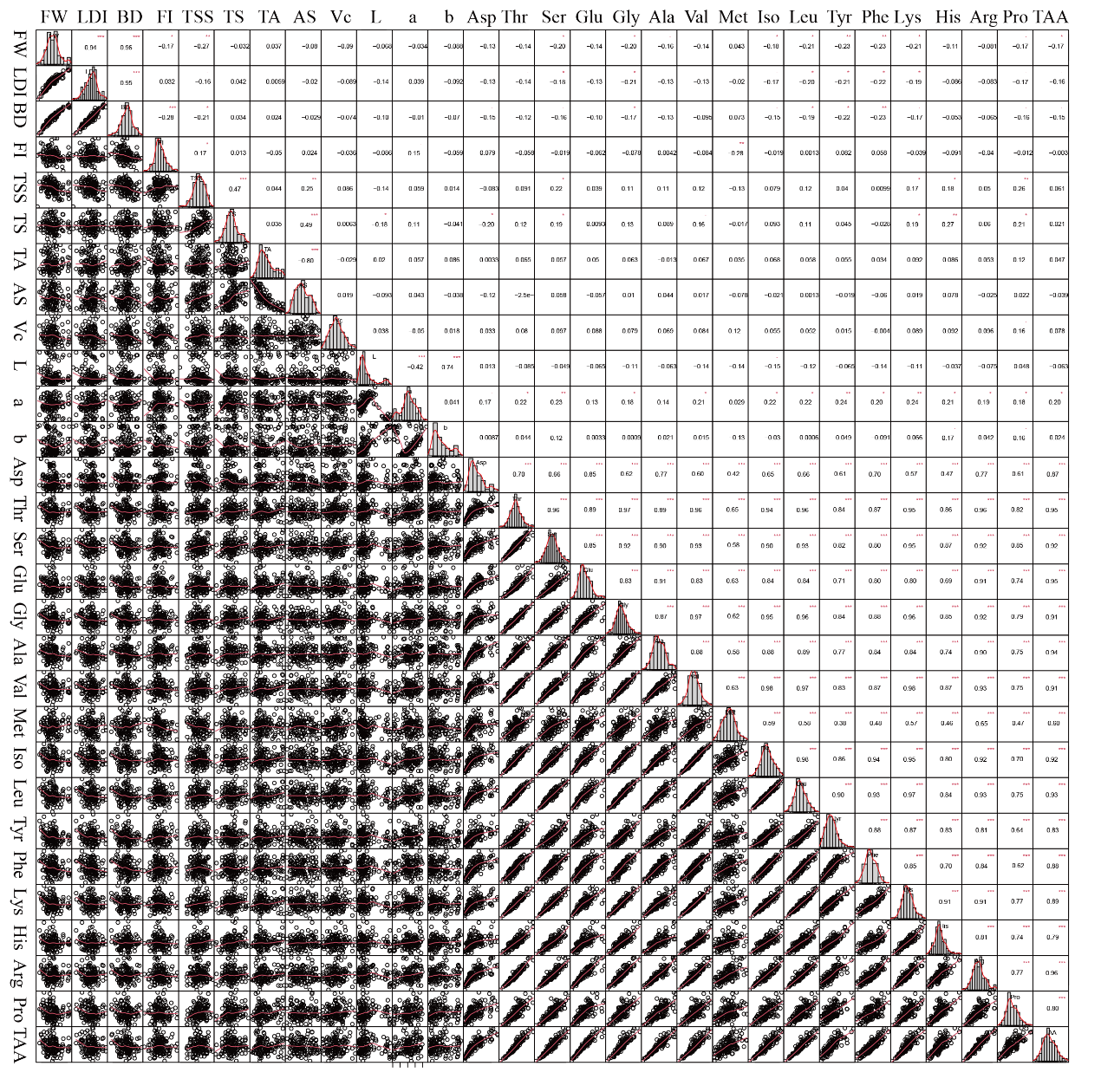


**Figure S7.** Correlation analysis, linear analysis, and normal distribution of 29 phenotype traits.


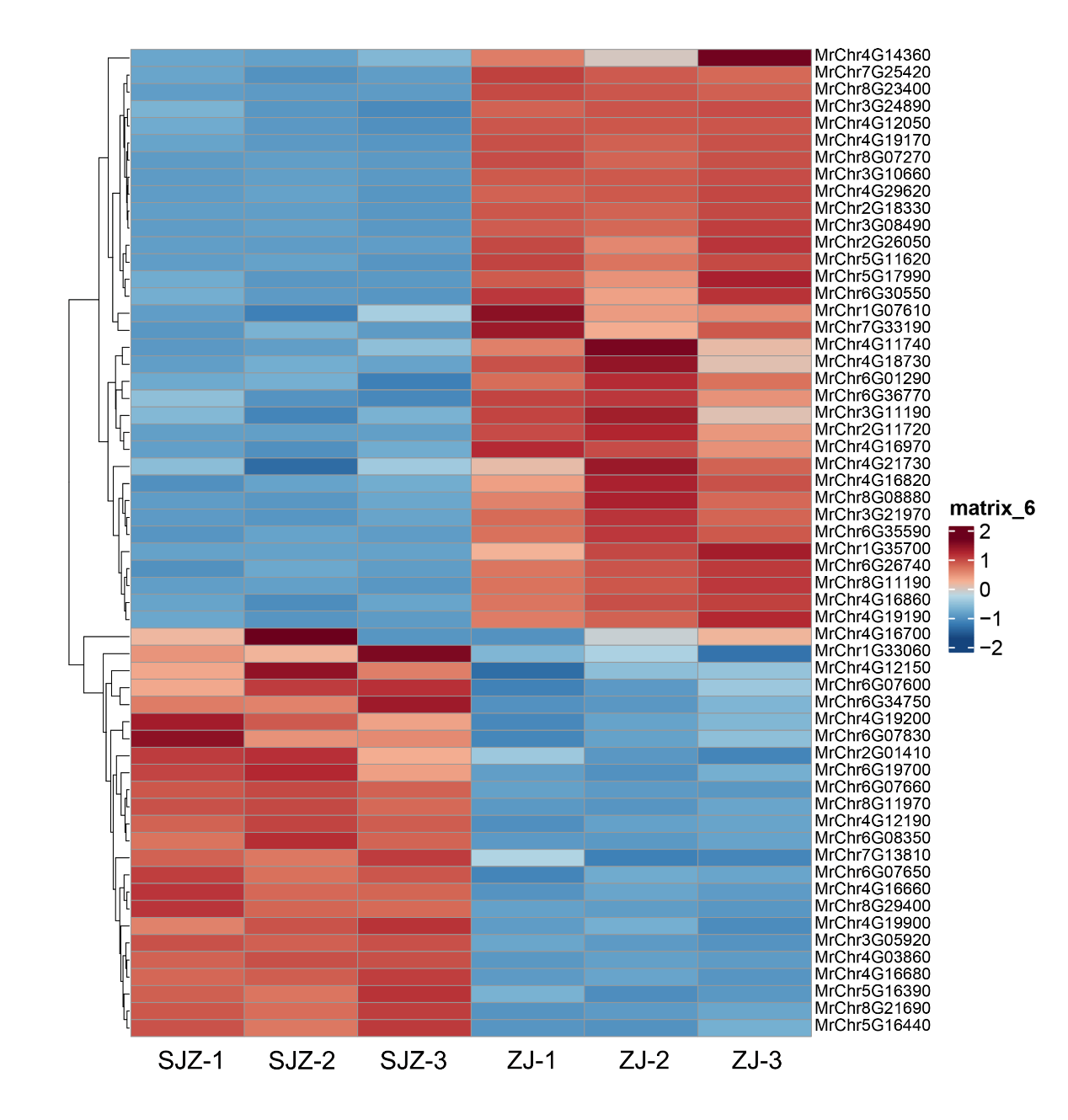


**Figure S8.** Fruit color-related DEGs from SJZ and ZJ fruit. SJZ-1, SJZ-2, and SJZ-3 were triple-replicate samples of SJZ fruit, ZJ-1, ZJ-2, and ZJ-3 were triple-replicate samples of ZJ fruit.


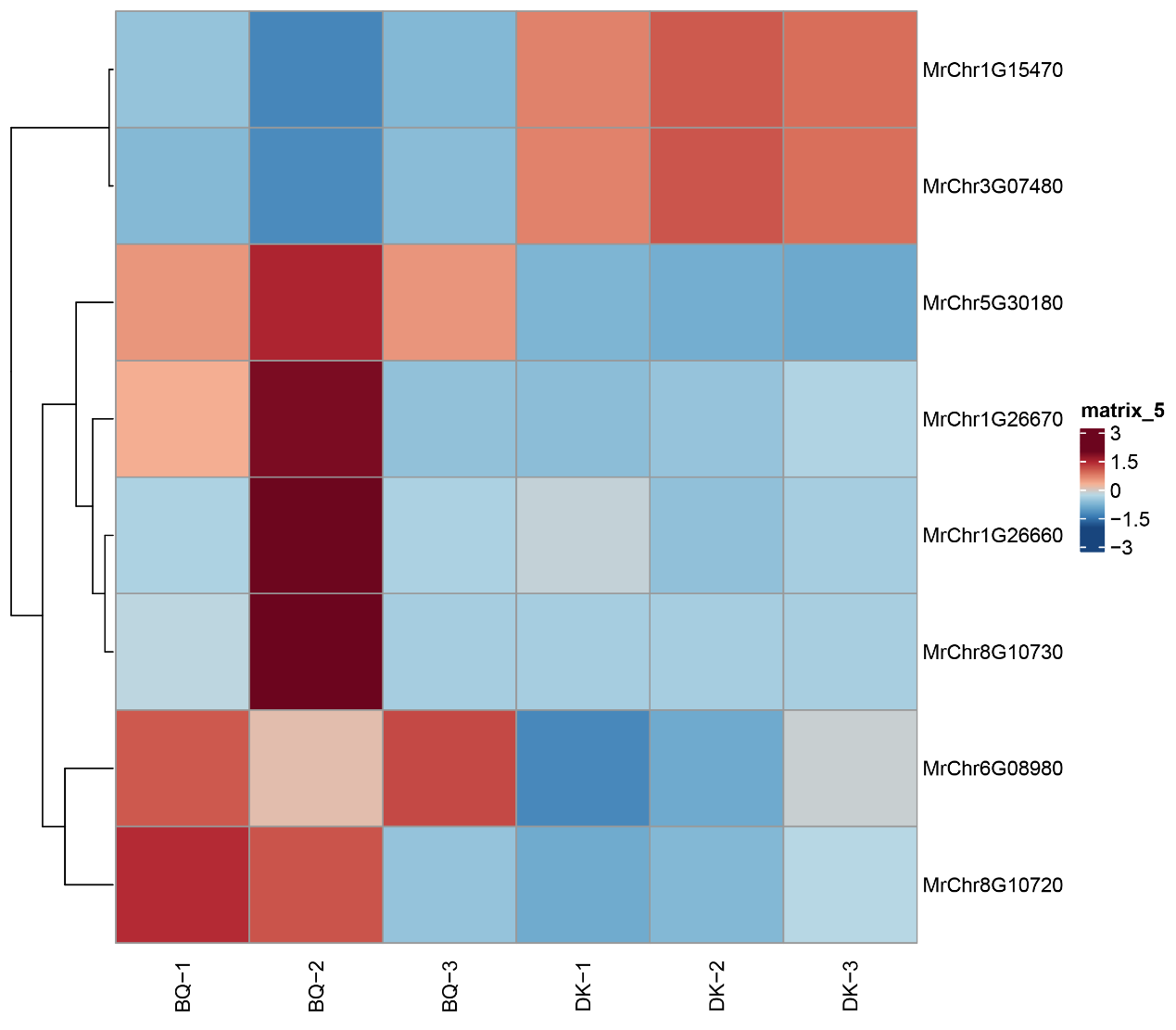


**Figure S9.** Fruit size-related DEGs from DK and BQ fruit. DK-1, DK-2, and DK-3 are triple-replicate samples of DK fruit, BQ-1, BQ-2, and BQ-3 are triple-replicate samples of BQ fruit.


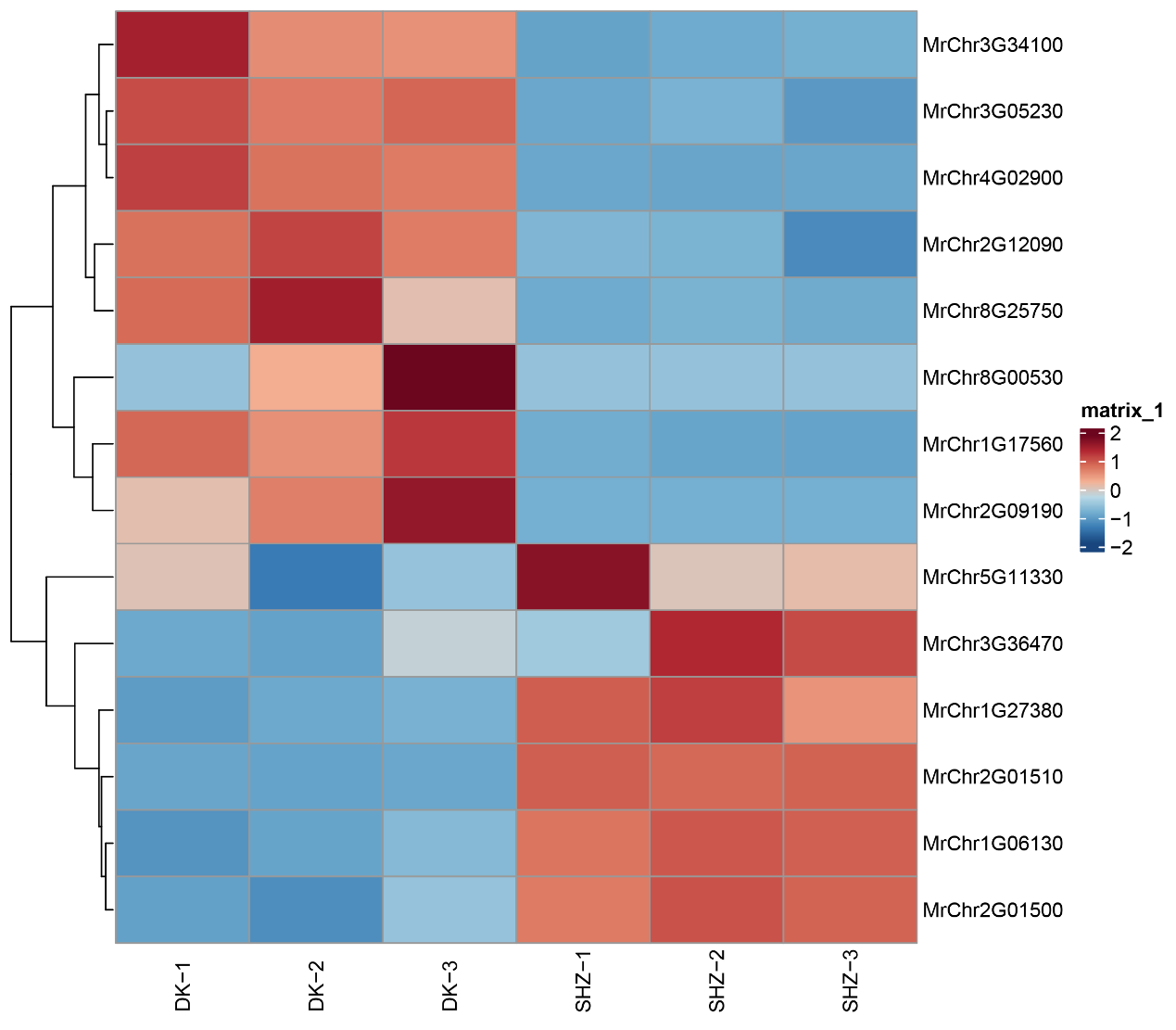


**Figure S10.** DEGs associated with fruit sugar and acid traits from DK and SHZ fruit. DK-1, DK-2, and DK-3 are triple-replicate samples of DK fruit, SHZ-1, SHZ-2, and SHZ-3 are triple-replicate samples of SHZ fruit.


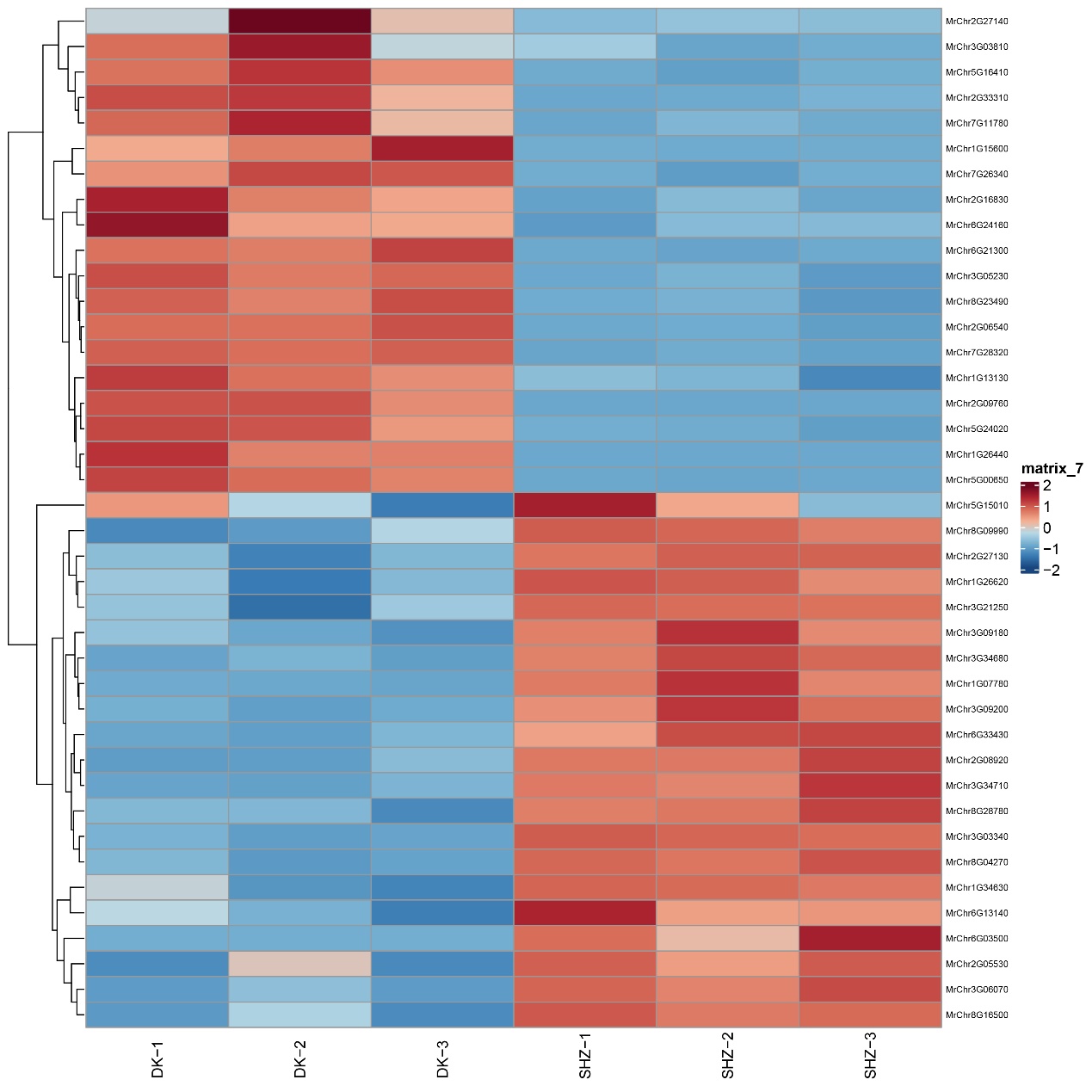


**Figure S11**. DEGs associated with amino acids from DK and SHZ fruit. DK-1, DK-2, and DK-3 are triple-replicate samples of DK fruit, SHZ-1, SHZ-2, and SHZ-3 are triple-replicate samples of SHZ fruit.
